# Heterogeneous nuclear ribonucleoprotein A1 (hnRNPA1) maintains muscle progenitor identity by stabilizing the *Ppp1r1b-lncRNA*–PRC2 complex

**DOI:** 10.1093/nar/gkag497

**Published:** 2026-05-19

**Authors:** Xuedong Kang, Yan Zhao, Stanley F Nelson, April Pyle, Aldons J Lusis, Marlin Touma

**Affiliations:** Department of Pediatrics, David Geffen School of Medicine, University of California, Los Angeles, CA 90095,United States; Neonatal/Congenital Heart Laboratory, Cardiovascular Research Laboratory, David Geffen School of Medicine, University of California, Los Angeles, CA 90095, United States; Department of Medicine, David Geffen School of Medicine, University of California, Los Angeles, CA 90095, United States; Department of Pediatrics, David Geffen School of Medicine, University of California, Los Angeles, CA 90095,United States; Department of Human Genetics, David Geffen School of Medicine, University of California, Los Angeles, CA 90095, United States; Microbiology, Immunology & Molecular Genetics, David Geffen School of Medicine, University of California, Los Angeles, CA 90095, United States; Department of Medicine, David Geffen School of Medicine, University of California, Los Angeles, CA 90095, United States; Department of Human Genetics, David Geffen School of Medicine, University of California, Los Angeles, CA 90095, United States; Microbiology, Immunology & Molecular Genetics, David Geffen School of Medicine, University of California, Los Angeles, CA 90095, United States; Department of Pediatrics, David Geffen School of Medicine, University of California, Los Angeles, CA 90095,United States; Neonatal/Congenital Heart Laboratory, Cardiovascular Research Laboratory, David Geffen School of Medicine, University of California, Los Angeles, CA 90095, United States

## Abstract

Heterogeneous nuclear ribonucleoprotein A1 (hnRNPA1) is a multifunctional RNA-binding protein of the hnRNP family, yet its role in long non-coding RNA (lncRNA)-mediated epigenetic regulation during myogenesis remains unclear. The lncRNA *Ppp1r1b-lncRNA* is an established regulator of myogenesis that functions through interaction with Polycomb repressive complex 2 (PRC2) at myogenic gene promoters. Here, we investigated the role of hnRNPA1 in *Ppp1r1b-lncRNA*-mediated regulation of myogenesis. Fluorescence *in situ* hybridization (FISH) revealed the subcellular localization of *Ppp1r1b-lncRNA*, and RNA pulldown coupled with mass spectrometry identified associated proteins. Both hnRNPA1 and EZH2 were found to bind *Ppp1r1b-lncRNA*, but at distinct regions. Knockdown of hnRNPA1 in mouse C2C12 myoblasts reduced the interaction between *Ppp1r1b-lncRNA* and EZH2, as determined by RNA immunoprecipitation (RIP), decreased promoter occupancy of *Ppp1r1b-lncRNA*, as assessed by chromatin isolation by RNA purification (CHIRP), and reduced H3K27me3 levels at the *MyoD1* and *Myogenin* promoters, as shown by chromatin immunoprecipitation (ChIP). These changes led to increased expression of muscle-specific transcription factors and sarcomeric genes, thereby disrupting the undifferentiated state. Furthermore, hnRNPA1 knockdown disrupted the interaction between the human ortholog *PPP1R1B-lncRNA* and PRC2 in human skeletal muscle precursor cells (hSMPCs). Together, these findings demonstrate that hnRNPA1 maintains the integrity of the *Ppp1r1b-lncRNA*–PRC2 complex and ensures proper epigenetic regulation of myogenic gene expression. This conserved hnRNPA1–*Ppp1r1b-lncRNA*–PRC2 regulatory axis represents a potential therapeutic target for muscle regeneration.

## Graphical Abstract

**Graphical Abstract:**
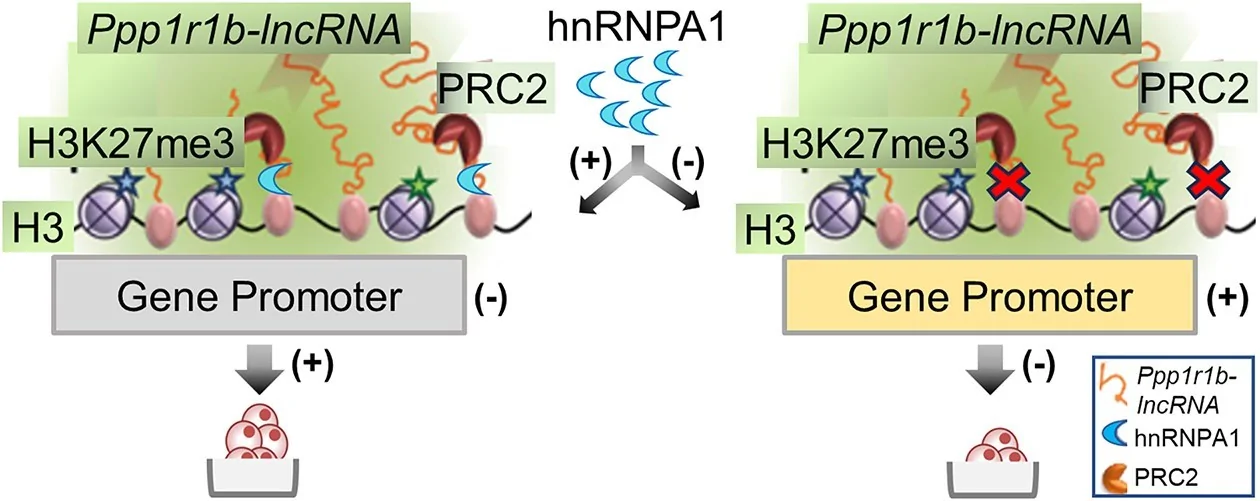

## Introduction

Muscle development during embryogenesis and regeneration relies on the coordinated expression of genes that control the transition from proliferation to differentiation. This transition requires extensive remodeling of the transcriptome, epigenetic landscape, and chromatin organization [1]. We have previously shown that *Ppp1r1b-lncRNA* is essential for normal myogenesis, as its knockdown reduced expression of myogenic transcription factors and blocked the differentiation of cardiac and skeletal myocytes. We further demonstrated that *Ppp1r1b-lncRNA* interacts with Polycomb repressive complex 2 (PRC2) and myogenesis gene promoters, and regulates myogenic differentiation via epigenetic mechanisms [2]

Long non-coding RNAs (lncRNAs) regulate gene expression through interactions with DNA, RNA, and proteins, acting in a dosage- and context-dependent manner [3, 4]. At low levels, they may stabilize core protein complexes and exert baseline regulatory functions, whereas higher expression can alter subcellular distribution, recruit accessory factors, and modify protein function [5–7]. Nuclear enrichment favors chromatin remodeling and epigenetic modification, while cytoplasmic enrichment enhances microRNA (miRNA) sponging, mRNA stability, and translational control. As a result, the biological outcomes of lncRNA activity are highly non-linear, as exemplified by MALAT1, H19, and NEAT1, which exert distinct and even opposing functions depending on expression level and cellular context [8].

Heterogeneous nuclear ribonucleoprotein A1 (hnRNPA1) is an RNA-binding protein (RBP) involved in splicing, mRNA stability, and translation [9]. hnRNPA1 knockout mice display lethal muscle differentiation defects and dilated cardiomyopathy, and reduced hnRNPA1 expression disrupts cardiac transcriptional networks, underscoring its critical role in muscle and heart development [10]. Pathogenic variants in *HNRNPA1* have been associated with congenital heart defects and amyotrophic lateral sclerosis (ALS) [11, 12]. Previous reports suggested that certain lncRNAs, such as Xist, recruit PRC2/PRC1 via hnRNPK–RBP interactions [13], but no study to date has directly investigated the functional involvement of hnRNPA1 in lncRNA-mediated myogenesis.

A precise balance between the progenitor pools and terminally differentiated populations is essential for functional myogenesis. Regulation of the proliferation-to-differentiation transitions is crucial for preserving a pool of undifferentiated cells and ensuring proper timing of differentiation [1]. Here, we identify an hnRNPA1–*Ppp1r1b-lncRNA*–PRC2 complex and investigate its role in maintaining the undifferentiated state of myoblasts, providing new mechanistic insight into lncRNA-based control of muscle development.

## Materials and methods

### Cell culture and treatment

C2C12 cells were obtained from the American Type Culture Collection (ATCC) and cultured in Dulbecco’s modified Eagle’s medium (DMEM), 10% fetal bovine serum (FBS), and 1% penicillin–streptomycin solution at 37°C, with 5% CO_2_. Human skeletal muscle precursor cells (hSMPCs), derived from human induced pluripotent stem cells (hiPSCs), were obtained from the UCLA center for Duchenne muscular dystrophy, Los Angeles, CA, United States. Cells were cultured according to protocols established at the April Pyle lab [14]. Cell passaging was performed when cell density approached 80% for subculture and 40–50% for transfection of small interfering RNA (siRNA). For differentiation, the growth medium was replaced with differentiation medium.

### Fluorescence *in situ* hybridization

The fluorescence *in situ* hybridization (FISH) Tag™ RNA Multicolor Kit (Thermo Fisher, F32956) was used. The *Ppp1r1b-lncRNA* gene was cloned into plasmid pCMV-Sport 6.1 between the T7 and SP6 promoters. The T7 polymerase generated an antisense RNA probe complementary to the target that hybridized to the lncRNA. The SP6 RNA polymerase generated a sense RNA probe that did not hybridize to the lncRNA and served as a negative control. The *Ppp1r1b-lncRNA* probe was labeled with Alexa Fluor™ 555 and used as a probe to identify the naturally occurring position of endogenous *Ppp1r1b- lncRNA* sequences *in situ* in fixed C2C12 samples. Cells were fixed in 4% (v/v) formaldehyde in phosphate-buffered saline (PBS) for 20 min. After rinsing with PBT, the cells were pre-hybridized with 100 µg ml^–1^ fragmented salmon testes DNA and rinsed. After incubating in hybridization buffer at 55°C for 5 min, the cells were incubated overnight with probe/hybridization buffer and washed with hybridization buffer and PBT. The cells were then incubated overnight with primary antibody and appropriate AlexaFluor-conjugated secondary antibodies for 1 h. Cell nuclei were eventually counterstained by 4′,6-diamidino-2-phenylindole (DAPI). Images were recorded on an echo revolve microscope (Discover Echo Inc., San Diego, CA, United States).

### Real-time PCR analysis

Total RNA was isolated from the cultured cells using the PureLink™ RNA Mini Kit (Thermo Fisher Scientific, 12183025). The reverse transcription reaction was performed with 1 µg of total RNA and the High-Capacity cDNA Reverse Transcription Kit (Thermo Fisher Scientific, 4368814), and the real-time PCRs were performed by using iTaq Universal SYBR Green Supermix (BioRad, 1725121). Primers were prepared by Integrated DNA Technologies. The relative expression value was calculated using the comparative threshold cycle (ΔΔCT) method. RNA primer sequences are listed in Supplementary Table S1.

### Western blot analysis

Cell extracts from cultured cells were denatured by bringing them to 95°C for 5 min with 4× sodium dodecylsulfate (SDS) loading buffer in proportion. Denatured protein (20 mg) was loaded and separated on an 8–16% SDS–polyacrylamide gel. After electrophoresis, the iBlot 2 Dry Blotting System (Thermo Fisher Scientific, IB21001) and iBind Western System (Thermo Fisher Scientific, SLF2000) were used to transfer proteins to a polyvinylidene fluoride (PVDF) membrane for analysis with antibodies. Blots were developed using SuperSignal™ West Pico PLUS Chemiluminescent Substrate (Life Technologies, 34580). Antibodies and their sources are listed in Supplementary Table S2.

### Chromatin isolation by RNA purification

The Magna chromatin isolation by RNA purification (CHIRP) RNA interactome kit (Millipore Sigma, 17-10494) was used. Assays were performed according to the manufacturer’s protocol. Capture probes were designed to target *Ppp1r1b-lncRNA* and prepared at a 50 µM total oligo concentration. Cells were cross-linked in 1% glutaraldehyde/PBS and sonicated to shear DNA. Probes were added to the cell lysate and incubated overnight for hybridization. After hybridization, streptavidin magnetic beads were added and incubated for an additional 30 min. After washing, the beads were incubated with RNase A and RNase H in the DNA elution buffer. The eluted sample was then subjected to protease K treatment, DNA isolation, and quantitative PCR (qPCR) analysis. A LacZ probe set was used as a negative control to evaluate non-specific probe binding. Neat1 RNA was used as a positive control to validate the efficiency of the CHIRP assay (Supplementary Fig. S1A). CHIRP capture probes and their sequences are listed in Supplementary Table S3

### Chromatin immunoprecipitation

A chromatin immunoprecipitation (ChIP)-IT High Sensitivity^®^ kit was used (Active Motif, 53 040). Undifferentiated and differentiated C2C12 as well as hnRNPA1-siRNA-treated C2C12 cells were exposed to 1% formaldehyde, and chromatin was fragmented by sonication. Immunoprecipitation was performed using IgG (negative control) or with the primary antibodies for H3K27me3. DNA was purified from the immunoprecipitants using the PCR purification kit (Qiagen, 28 104). Subsequently, real-time PCR was performed to amplify the promoter region (sequence) DNA of Myogenin and MyoD1. Normal rabbit IgG was used as a negative control. Hoxa9, a known target locus of H3K27me3, was used as a positive control (Supplementary Fig. S1B).

### RNA immunoprecipitation

The Magna RIP® RNA-Binding Protein Immunoprecipitation Kit (Millipore Sigma, 170 700) was used. Cell lysate was incubated with Protein A/G beads–antibody complex and incubated overnight at 4°C with gentle rotation. After washing off unbound material, RNAs bound to immunoprecipitated EZH2 were isolated, and quantitative reverse transcription–PCR (qRT–PCR) was performed using primers for mouse *Ppp1r1b-lncRNA* or human *PPP1R1B-lncRNA*.

Normal rabbit IgG was used as a negative control to evaluate non-specific immunoprecipitation, and a known EZH2-associated lncRNA-Neat1 was used as a positive control (Supplementary Fig. S1C, D).

### RNA pulldown

The Pierce™ Magnetic RNA-Protein Pull-Down Kit was used (Thermo Scientific, 20 164). The assay was performed with C2C12 cell lysates. Riboprobe® System T7 and Riboprobe® System SP6 (Promega, P1440, P1420), and Biotin RNA labeling mix (Roche, 11 685 597 910) were used for the preparation of biotin-labeled *Ppp1r1b-lncRNA*. After incubating biotinylated RNA with cell extract at 4°C, washed Dynabeads M280 Streptavidin (Thermo Fisher Scientific, 11206D) were added to the pulldown mixture. After incubation at 4°C for 1 h, the beads were collected using the magnetic apparatus (Thermo Fisher Scientific, 12320D) and washed with the binding buffer. Subsequently, the co-precipitated proteins were eluted in the elution buffer for mass spectometry analysis or in the SDS sample buffer and fractionated by 8–16% gradient SDS–polyacrylamide gel electrophoresis (PAGE) for western analysis. *In vitro* transcribed antisense *Ppp1r1b-RNA* was used as a negative control to assess non-specific binding. AR RNA [3′-untranslated region (UTR) of the androgen receptor (AR) RNA] was used as a positive control to validate the performance of the assay (Supplementary Fig. S1E). The full-length *Ppp1r1b-lncRNA* sequence was based on Ensembl transcript ENSMUST00000152525.2 (435 nt). Deletion constructs were generated according to exon boundaries. Full sequences are provided in Supplementary Data File S1, and construct coordinates are summarized in Supplementary Table S4.

### hnRNPA1 knockdown

Transient transfection of cells with *hnRNPA1-siRNA* (Cell Signaling Technology, 7668S and 6568S) was performed in 12-well plates (30 pmol per well) using Lipofectamine 2000 reagent (Life Technologies, 11668027). Scramble was used as a negative control. The knockdown efficiency of hnRNPA1 was assessed in three independent biological replicates (Supplementary Fig. S2).

### Statistical analysis

All results were confirmed in three independent experiments for reproducibility. Data are reported as the mean ± standard error of the mean (SEM). Comparisons between two groups were made using a two-tailed unpaired Student’s *t*-test. A one-way analysis of variance (ANOVA) followed by a Tukey’s post-test was used for multiple group comparisons.

## Results

### Differential expression of *Ppp1r1b-lncRNA* during myogenesis and its corresponding effects

**Figure 1.**
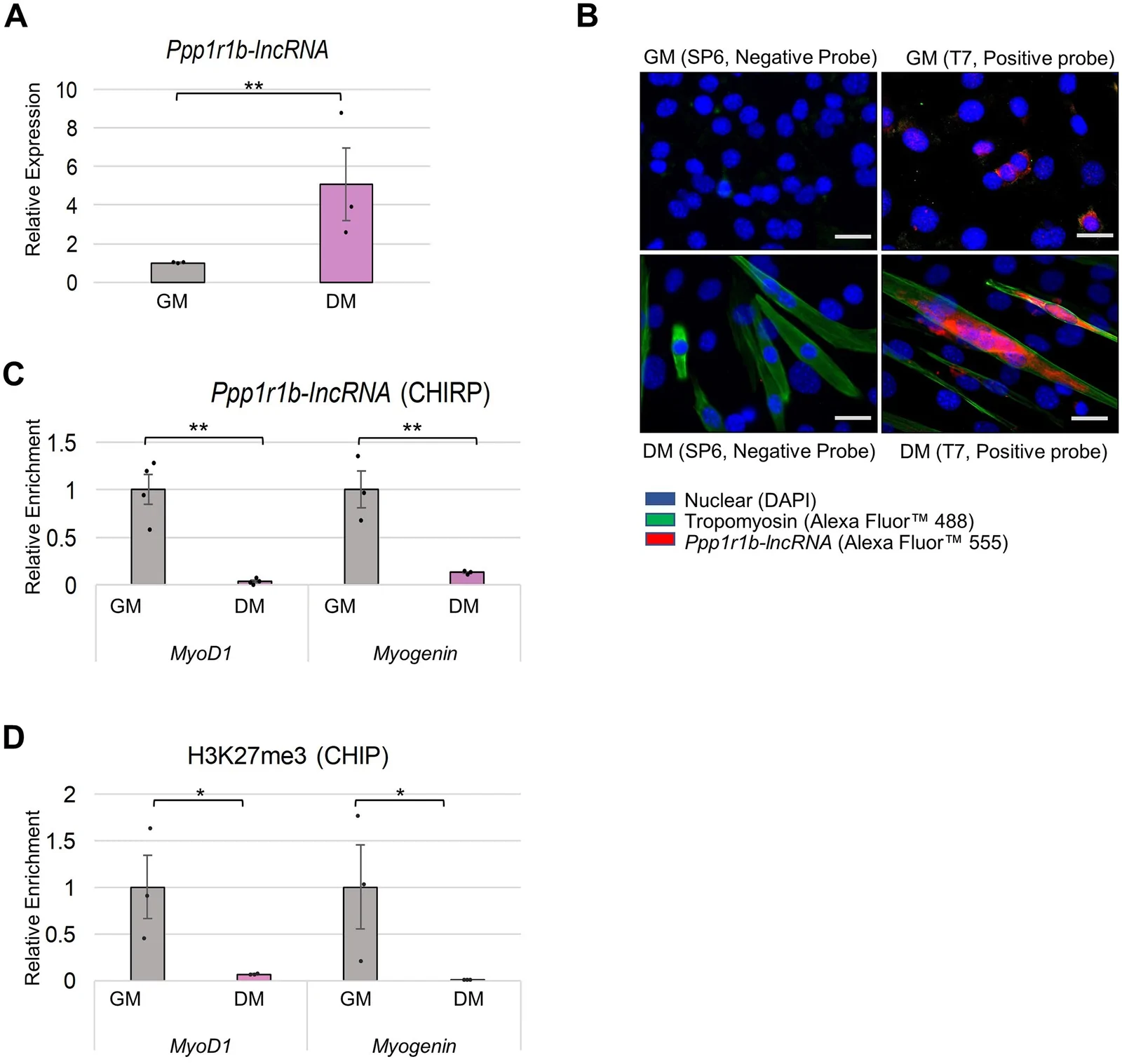
*Ppp1r1b-lncRNA* is up-regulated and displays changed subcellular localization, promoter occupancy, and histone 3 methylation during myocyte differentiation. (**A**) Real-time PCR analysis of *Ppp1r1b-lncRNA* expression before and after differentiation. (**B**) Subcellular localization of *Ppp1r1b-lncRNA* in myoblasts and differentiated cells was analyzed by FISH. (**C**) Interactions of *Ppp1r1b-lncRNA* with *MyoD1* and *Myogenin* promoter DNA were identified by CHIRP assays. (**D**) The abundance of histone modifications at the promoter regions of the myogenic genes was analyzed by ChIP assay using anti-H3K27me3 antibodies. GM, growth medium; DM, differentiation medium. Data are presented as the mean ± SEM from *n* = 3 independent biological replicates. Statistical significance was determined using an unpaired two-tailed Student’s *t*-test. *P < 0.05; **P < 0.01. Positive controls validating CHIRP and ChIP data are shown in Supplementary Fig. S1.

To determine the localization of *Ppp1r1b-lncRNA* during myogenic differentiation, C2C12, an immortalized mouse myoblast cell line, was used. We cultured C2C12 cells in high serum conditions for rapid proliferation and in low serum conditions for differentiation. The expression of *Ppp1r1b-lncRNA* in differentiated C2C12 cells was significantly higher than that in the undifferentiated cells (Fig. 1A). *Ppp1r1b-lncRNA* target *in situ* was detected by FISH. In undifferenced cells, *Ppp1r1b-lncRNA* was localized primarily in the nucleus and perinuclear cytoplasm, whereas in differentiated cells it was detected in both the nucleus and throughout most of the cytoplasm (Fig. 1B). Although *Ppp1r1b-lncRNA* expression is up-regulated during C2C12 differentiation, CHIRP assay revealed that its occupancy on the promoters of two myogenic factors, MyoD1 and Myogenin, was significantly reduced upon differentiation (Fig. 1C) accompanied by a decrease in H3K27me3 levels (Fig. 1D). These findings suggest that the function of *Ppp1r1b-lncRNA* may be modulated in a dosage-dependent manner. At lower levels in undifferentiated myoblasts, *Ppp1r1b-lncRNA* recruited the PRC2 complex to DNA to mediate epigenetic modifications. At higher levels during the differentiating process, *Ppp1r1b-lncRNA* promotes myogenic differentiation by competing for PRC2 binding [2]. Alternatively, distinct regulatory mechanisms or factors present in undifferentiated versus differentiated cells may contribute to the observed *lncRNA*–chromatin interactions.

Identification of the protein components in the *Ppp1r1b-lncRNA*–protein complex

**Figure 2.**
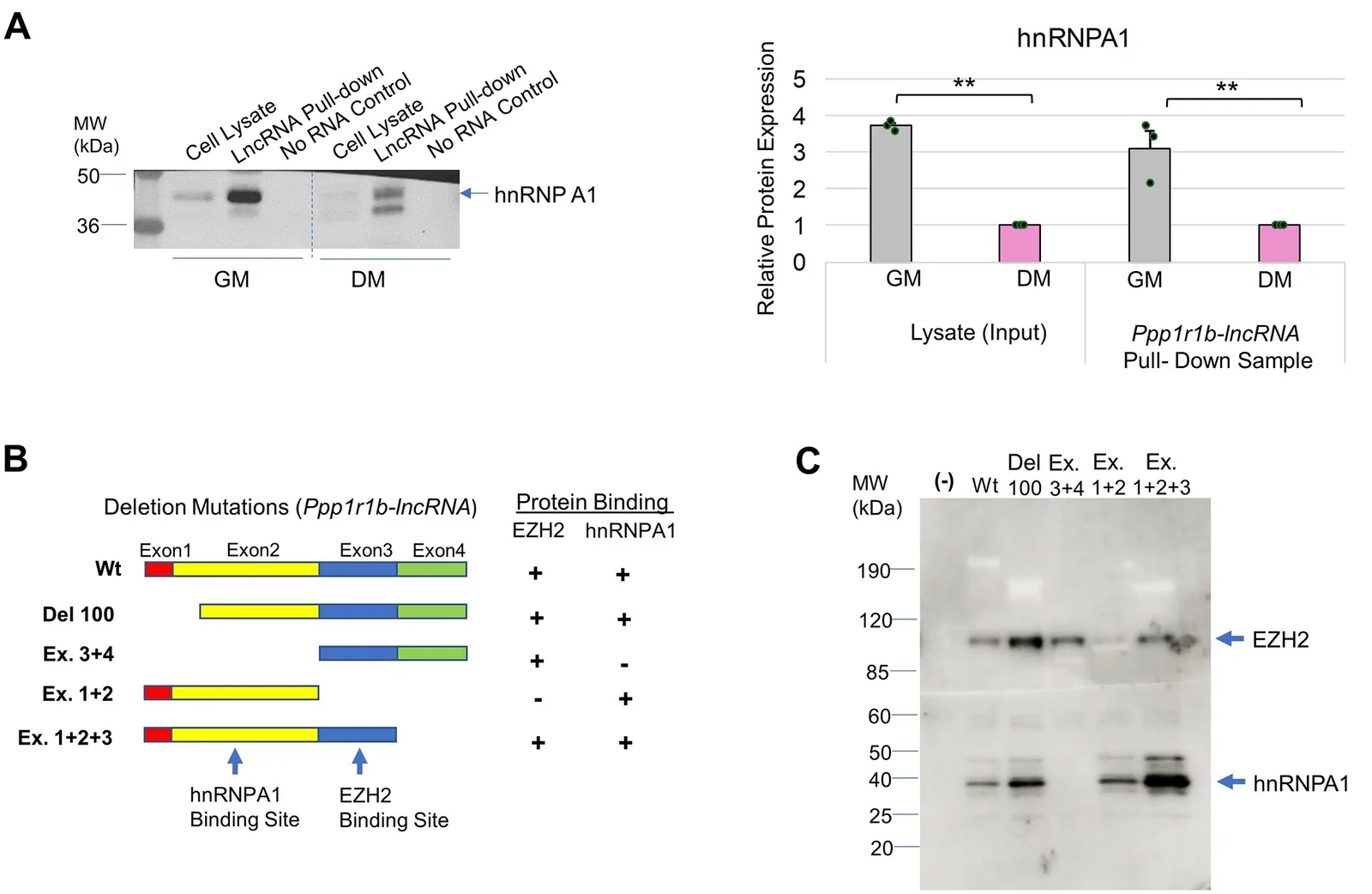
*Ppp1r1b-lncRNA* interacts with EZH2 and hnRNPA1 in a sequence-specific manner. RNA pulldown assays were performed using biotin-labeled *Ppp1r1b-lncRNA*. A scrambled RNA probe served as a negative control. (**A**) Western blotting of hnRNPA1 in cell lysate and the precipitate of *Ppp1r1b-lncRNA* pulldown from undifferentiated and differentiated C2C12 cells. The right panel shows densitometric quantification of hnRNPA1 levels in the input lysate and in RNA pulldown samples normalized to β-actin and the negative control. AR RNA was used as a positive control (see Supplementary Fig. S1). Data are presented as the mean ± SD from three independent experiments. Statistical significance was determined using an unpaired two-tailed Student’s *t*-test. *P*-value: **≤ 0.01; *≤ 0.05. (**B**) The full-length mouse *Ppp1r1b-lncRNA* gene was dissected and *in vitro* transcribed in different combinations as bait for RNA pulldown assay. (**C**) The precipitated proteins were fractionated by SDS–PAGE and subjected to western blotting for EZH2 and hnRNPA1. GM, growth medium; DM, differentiation medium.

We identified the proteins that associate with the *Ppp1r1b-lncRNA* by performing an RNA pulldown assay and mass spectrometry analyses. Lysates from non-differentiated and differentiated C2C12 cells were incubated with biotin-labeled *Ppp1r1b-lncRNA* immobilized on streptavidin magnetic beads. Analysis of the *Ppp1r1b-lncRNA*–bound proteins demonstrates that *Ppp1r1b-lncRNA* forms a multi-protein complex containing both RNA-binding proteins (hnRNPs) (Table 1) and chromatin-associated proteins (histones and RBBP4) (Table 2). This association implies a scaffold function, linking the RNA processing machinery with chromatin regulation. Among the identified hnRNPs, hnRNPA1 was prioritized for further validation based on its consistent enrichment in the pulldown fraction, high peptide coverage, multiple unique peptides, and strong peptide spectrum match confidence, as well as its enrichment ratio in undifferentiated/differentiated cells, rather than reliance on a single metric (Table 1; Fig. 2A; Supplementary Fig. S3). These features indicate a high-confidence interaction with *Ppp1r1b-lncRNA*. Remarkably, the more enriched hnRNPA1 in the *Ppp1r1b-lncRNA*–protein complex in undifferentiated cells coincided with higher levels of *Ppp1r1b-lncRNA* occupancy on chromatin (Fig. 1C) and H3K27me3 content on promoters of myogenesis genes, *MyoD1* and *Myogenin* (Fig. 1D). These results indicate that hnRNPA1 may contribute to modulation of epigenetic modifiers, such as the PRC2 subunits by physical interaction with the *Ppp1r1b-lncRNA*, thereby safeguarding the undifferentiated state of muscle progenitors. In addition, previous studies have demonstrated that hnRNPA1 plays a critical role in embryonic muscle development and cardiac function [10, 11]. Together, these quantitative and biological considerations support hnRNPA1 as a highly valuable candidate protein for future mechanistic studies. In addition to hnRNPA1, several other candidate proteins were identified (Supplementary Fig. S4). Among these, hnRNPA3 was further validated with biological replicates (Supplementary Fig. S4A), whereas hnRNPH2 and hnRNPU were detected at the screening level and are presented as qualitative observations (Supplementary Fig. S4B, C).

**Table 1.** hnRNPs identified by mass spectrometry analysis as being differentially enriched in the RNA pulldown complex from undifferentiated versus differentiated C2C12 cells

							Abundance				
Gene symbol	Accession	Description	Sum PEP score	Coverage (%)	No. of unique peptides	Score Sequest HT:Sequest HT	DN	DR	GN	GR	DR/GR
Hnrnpk	B2M1R6	Isoform of P61979, heterogeneous nuclear ribonucleoprotein K	137.185	42	18	79.25	15.7	87.1	0	197.2	0.36
Hnrnpm	Q9D0E1	Heterogeneous nuclear ribonucleoprotein M	193.958	52	37	66.55	23.5	96	1.4	180.5	0.4
Hnrnpa3	Q8BG05-2	Isoform of Q8BG05, isoform 2 of heterogeneous nuclear ribonucleoprotein A3	206.533	40	2	137.2	1.2	88.8	0	210	0.42
Hnrnpl	G5E924	Isoform of Q8R081, heterogeneous nuclear ribonucleoprotein L (fragment)	107.57	32	16	38.65	10	96.1	0	193.9	0.44
Hnrnpa1	P49312	Heterogeneous nuclear ribonucleoprotein A1	223.128	53	14	167.82	5.1	99.5	0.2	195.5	0.48
Hnrnph1	Q8C2Q7	Isoform of O35737, heterogeneous nuclear ribonucleoprotein H	89.607	27	3	52.34	26.4	113.2	0	160.4	0.54
Hnrnpu	Q8VEK3	Heterogeneous nuclear ribonucleoprotein U	125.763	25	22	46.19	5.7	109.7	0	184.5	0.56
Hnrnpa2b1	O88569	Heterogeneous nuclear ribonucleoproteins A2/B1	278.832	60	17	204.68	3.6	122.7	0.1	173.7	0.69
Hnrnpa3	Q8BG05	Heterogeneous nuclear ribonucleoprotein A3	239.965	43	5	171.95	3.8	134.9	1	161.3	0.81
Hnrnpd	Q60668	Heterogeneous nuclear ribonucleoprotein D0	60.36	23	3	12.56	25.8	134.1	19.7	140.1	0.9
Syncrip	G3UZI2	Isoform of Q7TMK9, heterogeneous nuclear ribonucleoprotein Q	66.187	19	7	22.34	11.5	152.8	5.3	135.7	1.08
Hnrnpul2	Q00PI9	Heterogeneous nuclear ribonucleoprotein U-like protein 2	66.346	22	17	18.22	5	204.7	0	90.4	2.2
Hnrnpr	Q8VHM5	Heterogeneous nuclear ribonucleoprotein R	69.774	19	9	18.12	15.2	218.5	0.9	66.3	3.11
Hnrnph2	P70333	Heterogeneous nuclear ribonucleoprotein H2	80.066	27	2	49.37	1.9	243.6	0	56.4	4.29

DN, negative control of differentiated C2C12 cells; DR, RNA pulldown of differentiated C2C12 cells; GN, negative control of undifferentiated C2C12 cells; GR, RNA pulldown of undifferentiated C2C12 cells. The DR/GR ratio indicates protein enrichment (fold change) in differentiation medium (myotubes) relative to growth medium (myoblasts).

**Table 2.** Histones identified by mass spectrometry analysis were enriched in the RNA pulldown complex from undifferentiated versus differentiated C2C12 cells

							Abundance				
Gene symbol	Accession	Description	Sum PEP score	Coverage[%]	No. of unique peptides	Score Sequest HT:Sequest HT	DN	DR	GN	GR	DR/GR
Hist1h4a	P62806	Histone H4	52.842	51	6	37.15	18.8	113	12.7	168.2	0.61
Hist1h1a	P43275	Histone H1.1	47.235	33	5	20.25	0.7	131.3	0	168	0.78
Hist1h1c	P15864	Histone H1.2	67.033	42	4	26.99	3.9	140.2	0.1	155.9	0.87
Hist1h1b	P43276	Histone H1.5	43.55	34	7	14.49	13.1	139	2.6	147.9	0.85
Hist1h1e	P43274	Histone H1.4	57.349	32	3	22.6	4.6	236.5	0	59	3.93
Rbbp4	Q60972	Histone-binding protein RBBP4	28.011	16	3	3.89	79	126.4	84.6	94.5	4.79

DN, negative control of differentiated C2C12 cells; DR, RNA pulldown of differentiated C2C12 cells; GN, negative control of undifferentiated C2C12 cells; GR, RNA pulldown of undifferentiated C2C12 cells. The DR/GR ratio indicates protein enrichment (fold change) in differentiation medium (myotubes) relative to growth medium (myoblasts).

### hnRNPA1 and EZH2 both bind *Ppp1r1b-lncRNA* but at different regions

We previously demonstrated that *Ppp1r1b-lncRNA* interacts with EZH2 [2] and, in the present study, we further observed an association between *Ppp1r1b-lncRNA* and hnRNPA1. To examine which sequence regions mediate the interactions, and how these two proteins interact with each other, we generated partially deleted *Ppp1r1b-lncRNA* constructs and performed RNA pulldown assays (Fig. 2B; Supplementary Table S4). Deletion of the first 100 bp or exon 4 of *Ppp1r1b-lncRNA* did not impair the binding of either hnRNPA1 or EZH2. In contrast, deletion of both exons 1 and 2 disrupted the interaction between *Ppp1r1b-lncRNA* and hnRNPA1, while EZH2 binding remained unaffected. Conversely, deletion of exons 3 and 4 resulted in loss of detectable EZH2 binding but did not affect hnRNPA1 binding (Fig. 2C; Supplementary Fig. S5). These findings indicate that EZH2, hnRNPA1, and *Ppp1r1b-lncRNA* form a multi-component complex, and their interactions are sequence specific. Specifically, EZH2 binds to exon 3, whereas hnRNPA1 preferentially binds to exon 2, indicating that the two proteins interact with distinct regions of *Ppp1r1b-lncRNA*.

### hnRNPA1 regulates myogenic gene expression by modulating the interaction of *Ppp1r1b-lncRNA* with PRC2

**Figure 3.**
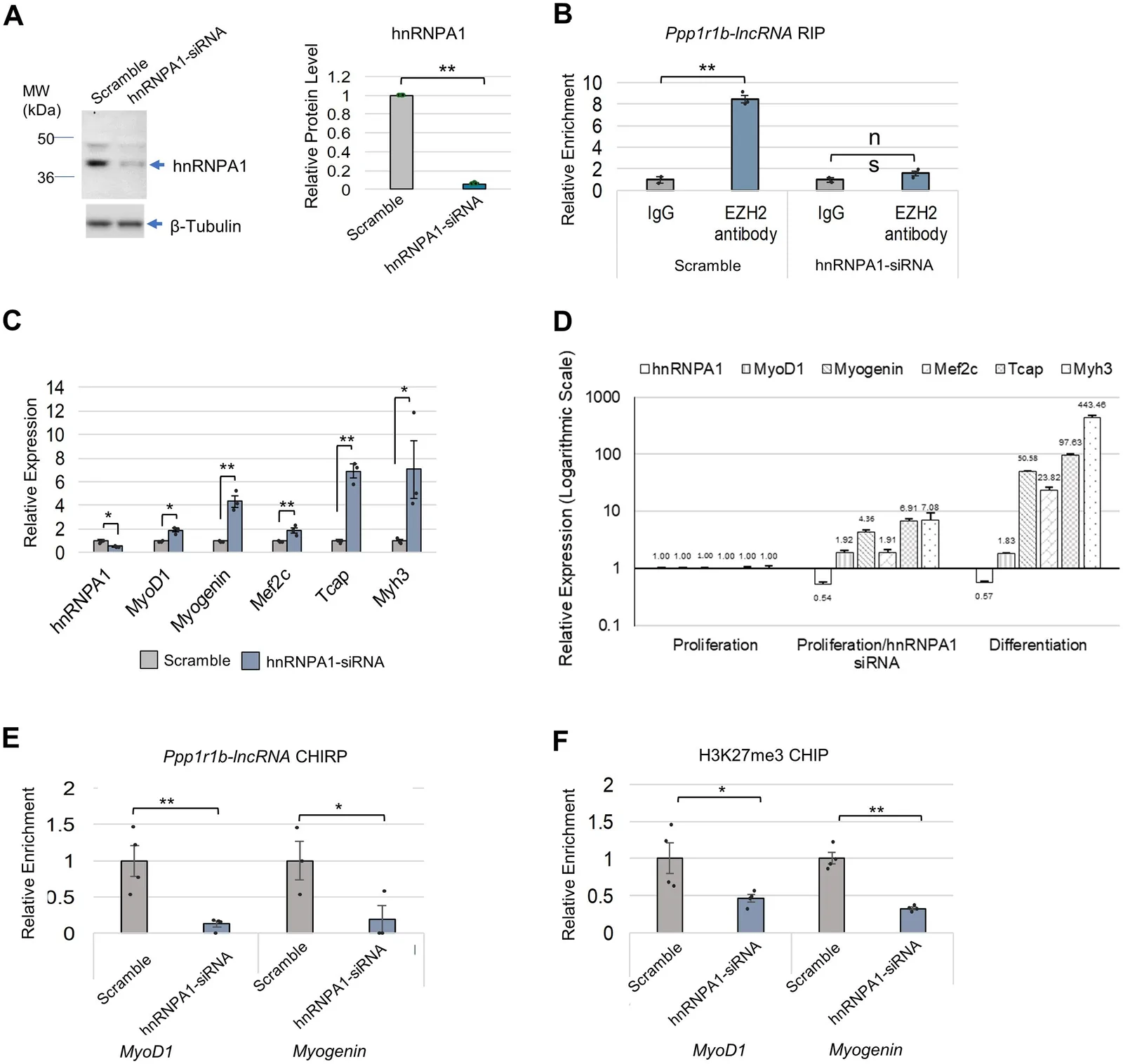
Down-regulation of hnRNPA1 disrupts the *Ppp1r1b-lncRNA*–PRC2 complex assembly, decreases *Ppp1r1b-lncRNA* occupancy and histone 3 methylation on myogenic gene promotors, and mitigates the expression of muscle-related genes in undifferentiated C2C12 cells. (**A**) Western blotting analysis for hnRNPA1 in C2C12 mouse myoblasts, cultured in growth medium, after siRNA treatment. The right panel shows densitometric quantification of hnRNPA1 protein levels normalized to the corresponding loading control from three independent biological replicates (Supplementary Fig. S2). (**B**) RIP assay analysis of *Ppp1r1b-lncRNA*–PRC2 complex assembly after hnRNPA1 suppression in C2C12 myoblasts (growth medium) using an EZH2 antibody. (**C**) Expression of myogenic transcriptional regulators and muscle-specific structural genes following hnRNPA1 depletion in C2C12 myoblasts (growth medium) was identified by RT–PCR. (**D**) RT–PCR analysis of myogenic transcriptional regulators and muscle-specific structural genes during proliferation, following hnRNPA1 depletion, and during normal differentiation. A logarithmic scale was used. (**E**) CHIRP assays reveal *Ppp1r1b-lncRNA* interactions with *MyoD1* and *Myogenin* promoter DNA in C2C12 myoblasts (growth medium) before and after hnRNPA1 suppression. (**F**) H3K27me3 CHIP assays reveal the abundance of the histone modifications at the promoter regions of the myogenic genes in C2C12 myoblasts (growth medium) before and after hnRNPA1 suppression using anti-H3K27me3 antibodies. All data from the siRNA treatment groups were normalized to the scramble control. Data are presented as the mean ± SD from three independent experiments. Statistical significance was determined using an unpaired two-tailed Student’s *t*-test. *P*-value: **≤ 0.01; *≤ 0.05.

Our results suggest that *Ppp1r1b-lncRNA* interacts directly with the EZH2-containing PRC2 complex in myocytes. To investigate the potential effect of hnRNPA1 knockdown on this interaction, we performed RNA immunoprecipitation (RIP) assays using *hnRNPA1-siRNA*-treated cells and an EZH2 antibody. We demonstrated that knockdown of hnRNPA1 (Fig. 3A; Supplementary Fig. S2) significantly reduced the association between *Ppp1r1b-lncRNA* and PRC2 (Fig. 3B). We next examined the impact of hnRNPA1 loss on the expression of muscle-specific transcription factors using qRT–PCR analysis. The mRNA levels of myogenesis regulators, such as *Myogenin, MyoD1*, and *Mef2c*, as well as muscle-specific structural genes, including *Tcap* and *Myh3*, were significantly elevated in hnRNPA1 siRNA-treated cells compared with untreated controls (Fig. 3C). The increased expression of transcription regulators and muscle-specific structural genes coincided with decreased EZH2 binding to *Ppp1r1b-lncRNA* (Fig. 3B). However, compared with normally differentiated cells, this increase in muscle-related gene expression following hnRNPA1 suppression was much lower (Fig. 3D), indicating impaired (inefficient) differentiation. Together, these results suggest that hnRNPA1 regulates myogenic gene expression by maintaining the stability of the *Ppp1r1b-lncRNA*–PRC2 complex on target gene promoters. The knockdown of hnRNPA1 was assessed in three independent biological replicates (Supplementary Fig. S2).

### hnRNPA1 modulates *Ppp1r1b-lncRNA* occupancy and histone methylation at muscle-specific transcription factor promoters

To further characterize the function of hnRNPA1 in *Ppp1r1b-lncRNA*-mediated myogenesis, we examined the effect of hnRNPA1 loss on histone methylation and *Ppp1r1b-lncRNA* occupancy at the promoters of muscle-specific transcription factors. Our data revealed that methylation levels at *Myogenin* and *MyoD1* promoters were significantly reduced in *hnRNPA1-siRNA*-treated cells compared with untreated controls (Fig. 3E). Consistently, hnRNPA1 knockdown led to a marked decrease in promoter occupancy of *Ppp1r1b-lncRNA* on these myogenic transcription factors (Fig. 3F).

### hnRNPA1 modulates the interaction of human *PPP1R1B-lncRNA* with the PRC2 complex in hSMPCs

**Figure 4.**
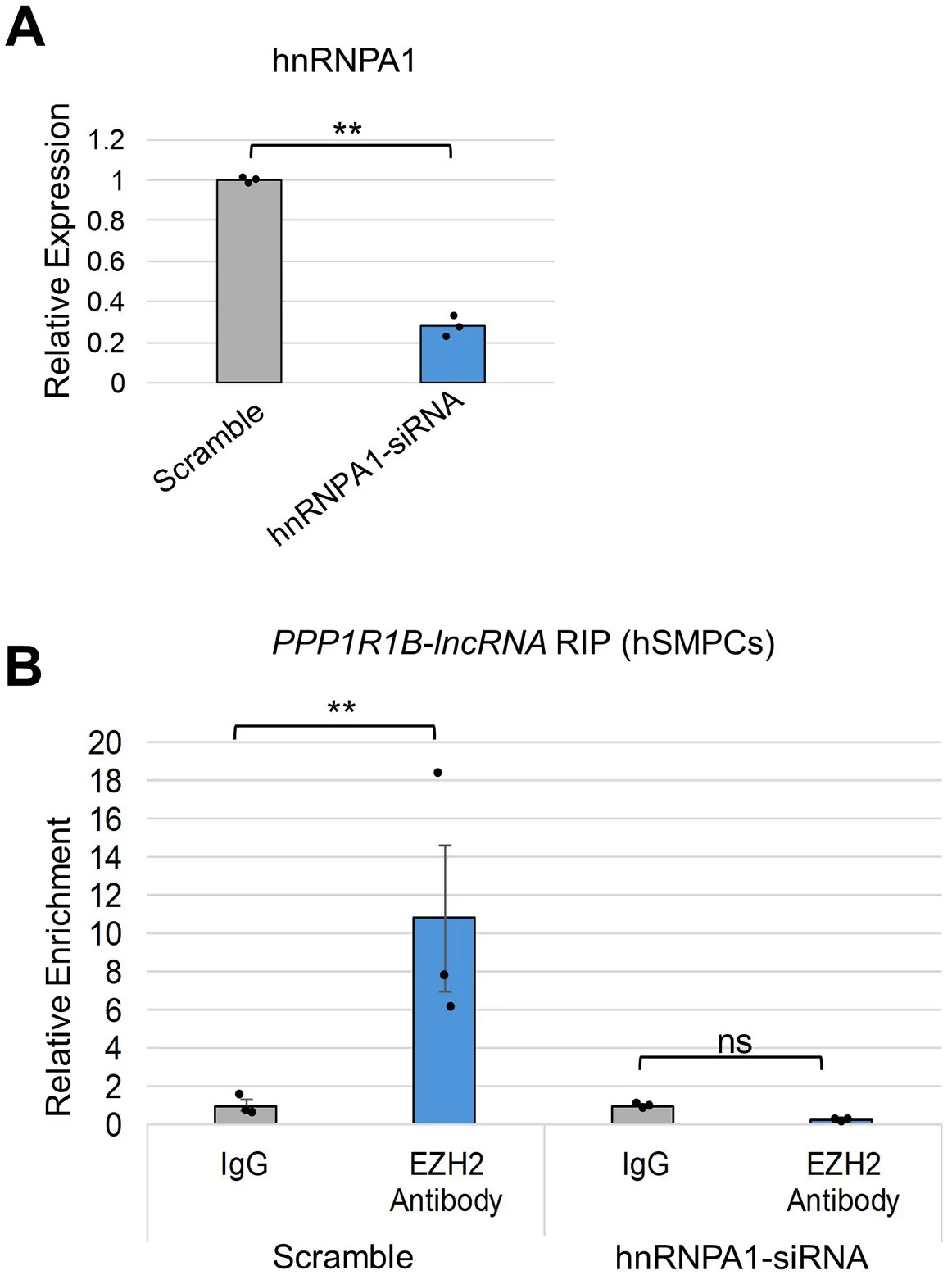
Down-regulation of hnRNPA1 disrupts the *PPPP1R1B-lncRNA*–PRC2 complex assembly in undifferentiated hSMPCs derived from hiPSCs. (**A**) RT–PCR analysis of hnRNPA1 expression following siRNA-mediated knockdown. (**B**) Effects of down-regulation of hnRNPA1 on assembly of the human *PPP1R1B-lncRNA*–PRC2 complex in undifferentiated hSMPCs was analyzed by RIP assay using an EZH2 antibody. Data are presented as the mean ± SEM from *n* = 3 independent biological replicates. Statistical significance was determined using an unpaired two-tailed Student’s *t*-test. *P < 0.05; **P < 0.01. See Supplementary Fig. S1 for positive controls.

Our previous work has demonstrated that the human ortholog, *PPP1R1B-lncRNA*, is also induced during myogenic differentiation, and that its knockdown leads to markedly impaired myotube formation. This impairment is accompanied by reduced myogenic master regulators and myosin protein levels compared with control cells. These findings indicate that *PPP1R1B-lncRNA* has a conserved function in human myogenesis [2]. To further determine the role of hnRNPA1 in the assembly of the human ortholog, *PPP1R1B-lncRNA*, with PRC2 in undifferentiated human cells, we performed a RIP assay using undifferentiated hSMPCs and an EZH2 antibody. The results showed that following hnRNPA1 knockdown, *PPP1R1B-lncRNA* was significantly reduced in the EZH2-pulled-down *PPP1R1B-lncRNA*–PRC2 complex (Fig. 4). These findings suggest that during human myogenesis, hnRNPA1 is also required for *PPP1R1B-lncRNA* interaction with PRC2, playing a conserved regulatory role in maintaining the undifferentiated state of muscle progenitor cells.

## Discussion

Dysregulation of hnRNPA1 has been linked to impaired muscle differentiation and certain muscle-related diseases [10, 11, 15, 16]. This study demonstrates that hnRNPA1 is an important component of the *Ppp1r1b-lncRNA*–protein complex that is required for stabilizing the *Ppp1r1b-lncRNA*–PRC2 complex assembly and maintenance of muscle progenitor cells. These findings establish a previously unrecognized regulatory axis linking RBPs, lncRNAs, and epigenetic machinery in myogenesis.

LncRNAs often show dosage- and location-dependent functions. At low expression levels, lncRNAs may fine-tune transcription or interact with a limited set of partners, whereas high levels can saturate binding sites or act as molecular sponges. Their subcellular localization is equally critical—nuclear lncRNAs typically regulate chromatin state, transcription, and splicing, while cytoplasmic lncRNAs modulate mRNA stability, translation, or signaling pathways. Some lncRNAs even shuttle between compartments, switching roles depending on cellular context. This combination of expression level and location explains why the same lncRNA can have distinct—even opposing—effects in different tissues or disease states [3, 17]. For example, the lncRNA TUNA is strongly expressed in embryonic stem cells (ESCs) to maintain Nanog and Sox2 transcription but becomes restricted to neural lineage cells, where it promotes neuronal differentiation rather than self-renewal [18]. Similarly, loss-of-function studies demonstrate that lncRNAs, such as Snhg3 and Gas5, are essential for pluripotency; their knockdown leads to loss of Nanog/Oct4/Sox2 expression, spontaneous differentiation, and reduced reprogramming efficiency [19].

Consistent with this context-dependent concept, our findings clearly demonstrate that both the expression level and subcellular localization of *Ppp1r1b-lncRNA* differ between undifferentiated and differentiated cells. In undifferentiated cells, *Ppp1r1b-lncRNA* exhibited a subcellular distribution predominantly within the nucleus and the perinuclear cytoplasmic region, whereas in differentiated cells it was detected in both the nucleus and diffusely throughout the cytoplasm, suggesting a redistribution of *Ppp1r1b-lncRNA* during myogenic differentiation and the operation of distinct regulatory mechanisms in these two cellular states. Our RNA pulldown and mass spectrometry analyses further revealed that *Ppp1r1b-lncRNA* assembles distinct protein complexes in proliferating versus differentiating cells, supporting a dosage- and location-dependent functional reprogramming model. The shift in expression level and localization probably remodels its protein interaction partners, thereby contributing to the transition from a repressive chromatin environment to one that permits myogenic gene activation.

Among the proteins identified, hnRNPA1 and EZH2 physically associate with *Ppp1r1b-lncRNA* at distinct regions, in a localization-dependent manner. Knockdown of hnRNPA1 significantly disrupts the *Ppp1r1b-lncRNA*–EZH2 complex in myoblasts from mice and humans, as shown by RIP assays, indicating that hnRNPA1 function is conserved and required for stable PRC2 recruitment by *Ppp1r1b-lncRNA* in living cells. Knockdown of hnRNPA1 also reduces PRC2 occupancy at myogenic promoters and decreases H3K27me3, resulting in transcriptional derepression of myogenic regulators. However, the transcript levels remain substantially lower than those observed in normally differentiated cells, indicating that hnRNPA1 depletion fails to fully activate the myogenic program. While these effects are largely PRC2 dependent, hnRNPA1 has diverse cellular roles—including RNA splicing, mRNA transport, and translational regulation [20]—suggesting that additional PRC2-independent mechanisms may contribute to the observed changes. Nevertheless, compared with normal differentiation, the increase in muscle differentiation-related gene expression following hnRNPA1 depletion can only be regarded as a perturbation of the stable state of myogenic progenitor cells.

Maintaining the proliferation of myoblasts and the stemness of their precursors (satellite cells) is critical for proper muscle development and regeneration. Stemness, marked by PAX7 expression, ensures that a pool of proliferative progenitors remains available until differentiation signals are present. Loss of stemness due to dysregulation of pathways, such as p38 MAPK, alterations in extracellular membrane stiffness, or niche disruption results in premature differentiation, progenitor depletion, and impaired myofiber formation [21, 22]. LncRNAs, such as SYISL, MALAT1, and MUNC, are key regulators of this quiescent state, recruiting PRC2, modulating transcriptional programs, and fine-tuning chromatin accessibility to prevent premature activation [23, 24]. Our results identify hnRNPA1 as a crucial factor for stabilizing the *Ppp1r1b-lncRNA*–PRC2 complex, thereby preserving the expansion capacity of myoblasts. By elucidating this regulatory axis, we provide new insight into the fine control of myogenic progenitor maintenance and offer potential therapeutic targets for enhancing muscle regeneration and combating conditions such as muscular dystrophies.

In comparison with well-characterized myogenic lncRNAs such as *MALAT1, H19*, and *NEAT1*, which regulate transcription or nuclear organization [6, 3], *Ppp1r1b-lncRNA* functions as a molecular scaffold stabilizing RNA–DNA–protein interactions within an epigenetic complex. Unlike lncRNAs such as *linc-MD1* and *Sirt1-AS*, which act through miRNA-related mechanisms [25, 26], or *Syisl*, which regulates gene expression via PRC2 recruitment [27], *Ppp1r1b-lncRNA* coordinates hnRNPA1 association with the PRC2 complex. This highlights its role in maintaining a repressive chromatin state in progenitor cells.

These findings also suggest potential therapeutic implications. Dysregulation of epigenetic control and impaired myogenic differentiation are hallmarks of multiple muscle disorders, including muscular dystrophies and age-related muscle degeneration [15, 21]. Targeting components of the *Ppp1r1b-lncRNA*–hnRNPA1–PRC2 axis may provide opportunities to modulate progenitor cell maintenance or enhance regenerative capacity. For example, strategies aimed at stabilizing this complex could help preserve stemness in degenerative conditions, whereas controlled disruption might promote differentiation in contexts where regeneration is impaired. However, these possibilities remain speculative, and further validation using *in vivo* and disease-relevant model systems will be necessary to evaluate therapeutic feasibility.

Importantly, the regulatory role of hnRNPA1 in this context appears to be evolutionarily conserved. Knockdown of hnRNPA1 significantly disrupts the *Ppp1r1b-lncRNA*–EZH2 complex in muscle progenitor cells from mice and humans, as shown by RIP assays, indicating that hnRNPA1 function is conserved and required for stable PRC2 recruitment by *Ppp1r1b-lncRNA* in living cells. This consistent effect of hnRNPA1 depletion in both mouse and human myoblasts supports a shared regulatory mechanism across species. Given the conserved RNA binding properties of hnRNPA1, and the emerging evidence for conserved lncRNA-mediated epigenetic regulation, this axis reflects a fundamental principle of muscle progenitor control. Such conservation enhances the translational relevance of our findings and supports their broader applicability to human muscle biology and disease.

Finally, several limitations of this study should be acknowledged. Our conclusions are primarily based on *in vitro* cell culture systems, which may not fully recapitulate the complexity of *in vivo* muscle development and regeneration. Additionally, although we demonstrate functional interactions among *Ppp1r1b-lncRNA*, hnRNPA1, and PRC2, the precise molecular mechanisms governing their dynamic assembly and regulation remain incompletely defined. Future studies using animal models and lineage-specific genetic approaches will be essential to validate the physiological relevance of this conserved regulatory axis and to further dissect its mechanistic details and translational applications.

## Conclusions and fututure directions

As an important component of the *Ppp1r1b-lncRNA*–protein complex, hnRNPA1 is required for efficient recruitment of the PRC2 complex by *Ppp1r1b-lncRNA* to the promoters of myogenic transcription factors in undifferentiated myoblasts. Loss of hnRNPA1 disrupts this recruitment, resulting in impaired promoter methylation and, probably, disturbance of baseline regulatory functions. Collectively, these findings provide critical insight into the functional relevance of the conserved hnRNPA1–*Ppp1r1b-lncRNA*–PRC2–promoter axis in muscle progenitor maintenance and regeneration. However, the full extent of hnRNPA1’s regulatory roles, including its interaction with *Ppp1r1b-lncRNA*, PRC2, and the *Myogenin* and *MyoD1* promoters during myogenesis, remains to be fully elucidated. Beyond hnRNPA1, our mass spectrometry analysis identified several other differentially associated proteins that may cooperate with *Ppp1r1b-lncRNA* in regulating chromatin and transcriptional states. Functional characterization of these interactors will provide a more comprehensive understanding of how the *Ppp1r1b-lncRNA*–protein interactome coordinates progenitor maintenance and myogenic differentiation during muscle development and regeneration.

## Supplementary Material

gkag497_Supplemental_Files

## Data Availability

Proteomic data has been uploaded in the PRIDE website under project accession number PXD072229. All data presented in this manuscript will be available for sharing with interested investigators.
