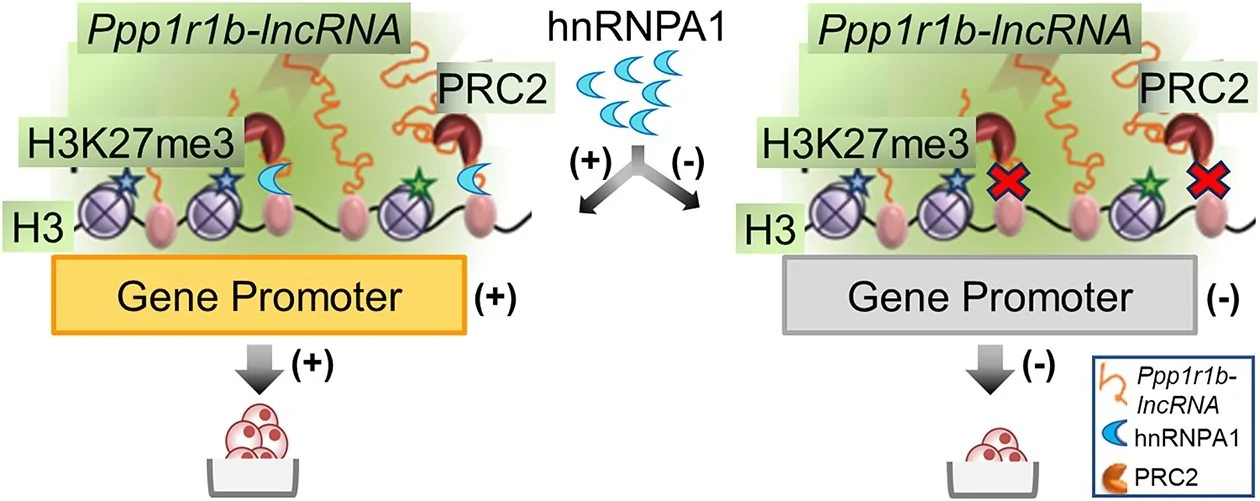# Correction to ‘Heterogeneous nuclear ribonucleoprotein A1 (hnRNPA1) maintains muscle progenitor identity by stabilizing the *Ppp1r1b-lncRNA*–PRC2 complex’

**DOI:** 10.1093/nar/gkag912

**Published:** 2026-09-23

This is a correction to: Xuedong Kang, Yan Zhao, Stanley F Nelson, April Pyle, Aldons J Lusis, Marlin Touma, Heterogeneous nuclear ribonucleoprotein A1 (hnRNPA1) maintains muscle progenitor identity by stabilizing the *Ppp1r1b-lncRNA*–PRC2 complex, *Nucleic Acids Research*, Volume 54, Issue 9, 22 May 2026, gkag497, https://doi.org/10.1093/nar/gkag497

In the originally published version of this manuscript, the graphical abstract contained errors in the annotation of the promoter elements.

The (+) and (−) signs displayed to the right of the Promoter Box were incorrectly assigned and have now been reversed in the corrected graphical abstract. The colours used to represent promoters have also been reversed in keeping with the scheme.

The corrected graphical abstract reads:

**Figure:**
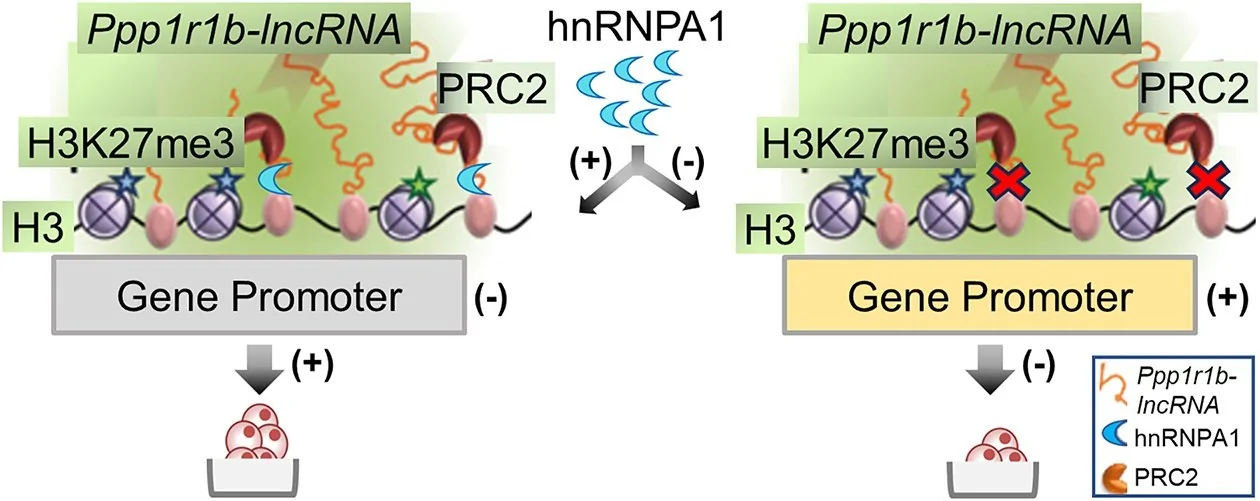


Instead of:

**Figure:**