# Supplementary material for: Heterogeneous nuclear ribonucleoprotein A1 (hnRNPA1) maintains muscle progenitor identity by stabilizing the Ppp1r1b-lncRNA–PRC2 complex
Source: Nucleic Acids Res. 2026 May 19;54(9):gkag497. doi: 10.1093/nar/gkag497 (PMC13183673; doi:10.1093/nar/gkag497)
Supplement: gkag497_Supplemental_Files [file gkag497_supplemental_files.zip › Supplementary Material.pdf]

Supplementary Data

Heterogeneous Nuclear Ribonucleoprotein A1 (hnRNPA1) Maintains Muscle Progenitor Identity by Stabilizing the *Ppp1r1b-lncRNA*-PRC2 Complex

Xuedong Kang[1,2], Yan Zhao[3], Stanley F. Nelson[1,4], April Pyle[5], Aldons J. Lusis [3,4,5], Marlin Touma*[1,2]

Authors' Affiliations

1. Department of Pediatrics, David Geffen School of Medicine, University of California, Los Angeles, CA.
2. Neonatal/Congenital Heart Laboratory, Cardiovascular Research Laboratory, David Geffen School of Medicine, University of California, Los Angeles, CA.
3. Department of Medicine, David Geffen School of Medicine, University of California, Los Angeles, CA
4. Department of Human Genetics, David Geffen School of Medicine, University of California, Los Angeles, CA.
5. Microbiology, Immunology & Molecular Genetics, David Geffen School of Medicine, University of California, Los Angeles, CA.

*Correspondence:
Marlin Touma, MD, PhD
Phone: 310.206.6197
Email: mtouma@mednet.ucla.edu

Supplemental Data File 1. *Ppp1r1b-lncRNA* sequences.

>WT_Ppp1r1b_lncRNA | ENSMUST00000152525.2 | 1–435 | 435 nt

GCCCACACTGTTCCTTTCCTCGGCTGCATCTGAGCAGCTGTGCAGCACCTGCAGACCATT

AGCAACTTGAGTGAGAACCAGGCCTCGGAGGAAGAGGATGAGTTAGGGGAGCTTCGGGAG

CTTGGGTACCCACAGGAGGATGATGAGGAGGATGAGGATGAAGAGGAGGACGAAGAAGAA

GACAGCCAGGCGGAGGTCCTGAAAGGCAGCAGGGGCACTGTGGGGCAGAAGCCTACTTGT

GGCCGGGGTCTGGAGGGGCCCTGGGAGCGCCCACCTCCTCTGGATGAGCCCCAGAGAGAT

GGAAACTCTGAGGACCAAGTGGAAGGCAGAGCAACACTAAGTGTCCTCTTTATTTGTCTT

TCAGAGCCTGGAGAGGAACCTCAGCATCCCAGCCCCCCCTGAGCCTGGCACATAAGCTCA

GAGCCCTGTATCTCC

>Del_1_100 | Δ1–100 nt | 101–435 | 335 nt

AGTAGGGGGAGCTTCGGGAGCTTGGGTACCCACAGGAGGATGATGAGGAGGATGAGGATGA

AGAGGAGGACGAAGAAGAAGACAGCCAGGCGGAGGTCCTGAAAGGCAGCAGGGGCACTGT

GGGGCAGAAGCCTACTTGTGGCCGGGGTCTGGAGGGGCCCTGGGAGCGCCCACCTCCTCT

GGATGAGCCCCAGAGAGATGGAAACTCTGAGGACCAAGTGGAAGGCAGAGCAACACTAAG

TGTCCTCTTTATTTGTCTTTCAGAGCCTGGAGAGGAACCTCAGCATCCCAGCCCCCCCTG

AGCCTGGCACATAAGCTCAGAGCCCTGTATCTCC

>Exon1_2 | 1–220 | 220 nt

GCCCACACTGTTCCTTTCCTCGGCTGCATCTGAGCAGCTGTGCAGCACCTGCAGACCATT

AGCAACTTGAGTGAGAACCAGGCCTCGGAGGAAGAGGATGAGTTAGGGGAGCTTCGGGAG

CTTGGGTACCCACAGGAGGATGATGAGGAGGATGAGGATGAAGAGGAGGACGAAGAAGAA

GACAGCCAGGCGGAGGTCCTGAAAGGCAGCAGGGG

>Exon3_4 | 221–435 | 215 nt

CACTGTGGGGCAGAAGCCTACTTGTGGCCGGGGTCTGGAGGGGCCCTGGGAGCGCCCACC
TCCTCTGGATGAGCCCCAGAGAGATGGAAACTCTGAGGACCAAGTGGAAGGCAGAGCAAC
ACTAAGTGTCCTCTTTATTTGTCTTTCAGAGCCTGGAGAGGAACCTCAGCATCCCAGCCC
CCCCTGAGCCTGGCACATAAGCTCAGAGCCCTGTATCTCC

>Exon1_2_3 | 1–343 | 343 nt

GCCCACACTGTTCCTTTCCTCGGCTGCATCTGAGCAGCTGTGCAGCACCTGCAGACCATT
AGCAACTTGAGTGAGAACCAGGCCTCGGAGGAAGAGGATGAGTTAGGGGAGCTTCGGGAG
CTTGGGTACCCACAGGAGGATGATGAGGAGGATGAGGATGAAGAGGAGGACGAAGAAGAA
GACAGCCAGGCGGAGGTCCTGAAAGGCAGCAGGGGCACTGTGGGGCAGAAGCCTACTTGT
GGCCGGGGTCTGGAGGGGCCCTGGGAGCGCCCACCTCCTCTGGATGAGCCCCAGAGAGAT
GGAAACTCTGAGGACCAAGTGGAAGGCAGAGCAACACTAAGTG

>Negative_control_antisense | antisense WT | 435 nt

GGAGATACAGGGCTCTGAGCTTATGTGCCAGGCTCAGGGGGGGCTGGGATGCTGAGGTTC
CTCTCCAGGCTCTGAAAGACAAATAAAGAGGACACTTAGTGTTGCTCTGCCTTCCACTTG
GTCCTCAGAGTTTCCATCTCTCTGGGGCTCATCCAGAGAGGAGGTGGGCGCTCCCAGGGC
CCCTCCAGACCCCGGCCACAAGTAGGCTTCTGCCCCACAGTGCCCCTGCTGCCTTTCAGG
ACCTCCGCCTGGCTGTCTTCTTCGTCCTCCTCTTCATCCTCATCCTCCTCATCATCCTCT
GGTACCCAAGCTCCCGAAGCTCCCCTAACTCATCCTCTTCCTCCGAGGCCTGGTTCTCAC
TCAAGTTGCTAATGGTCTGCAGGTGCTGCACAGCTGCTCAGATGCAGCCGAGGAAAGGAA
CAGTGTGGGC

Supplemental Tables

Table S1. List of primers used for quantitative RT-PCR

Primer	Species	Sequence (5'→3')
mPpp1r1b LncRNA	mouse	F: GCATCTGAGCAGCTGTGCAGCA
		R: CCTCCTCATCATCCTCCTGTGGGT
hPpp1r1b LncRNA	human	F: AAGGCTGGGGACCTTCCAACTGAAG
		R: GGAGGAGAGTTCACTTCGTGAGCCAC
mhnRNPA1	mouse	F:GAAACAACCGACGAGAGTCTG
		R: TGTGTGGTCTTGCATTCATG
hhnRNPA1	human	F:TCAGAGTCTCCTAAAGAGCCC
		R: ACCTTGTGTGGCCTTGCAT
mMyoD1	mouse	F: CCACTCCGGGACATAGACTTG
		R: AAAAGCGCAGGTCTGGTGAG
mMyogenin	mouse	F: GCAGGCTCAAGAAAGTGAATGA
		R: TAGGCGCTCAATGTACTGGAT
mMef2c	mouse	F: ATGGGGAGAAAAAAGATTCAGATTACGAG
		R: TGTACTTGAGCAACACCTTATCCATGTCA
mTcap	mouse	F: TGAGGAGAACCAGGAACGCAGGGA
		R: CCACAGGGGGAAGCTGCTTTGTGA
mMyh3	mouse	F: AAAAGGCCATCACTGACGC
		R: CAGCTCTCTGATCCGTGTCTC
mMyoD1 Promoter	mouse	F: GTCTCTCTGCCCTCCTTCCT
		R: CAAGCTCCGCCCTACTACAC
mMyogenin Promoter	mouse	F: GAGCCCCACTTCTATGATGG
		R: GAAGAAAAGGGACTGGGGAC

Table S2. List of antibodies and their sources

Antibody	Company	Catalog#	Application
Tropomyocin	Sigma-Aldrich	T9283	FISH
H3K27me3	Cell Signaling Technology	9733S	CHIP
hnRNPA1	Cell Signaling Technology	8443S	WB
EZH2	Cell Signaling Technology	5246S	WB/RIP
β-Tubulin	Thermo Fisher Scentific	MA5-16308	WB
Normal Rabbit IgG	Cell Signaling Technology	2729S	RIP/CHIP
β-Actin	Cell Signaling Technology	3700S	WB
hnRNPH2	Elabscience	E-AB-52276	WB
hnRNPA3	Elabscience	E-AB-19996	WB
hnRNPU	Abclonal Technology	A3917	WB
HuR	Thermo Fisher Scentific	1862775	WB

Table S3. List of CHIRP capture probes and their sequences

Probe Name	Sequence (5'→3')
mPpp1r1b207-probe1	TGCACAGCTGCTCAGATG/3BioTEG
mPpp1r1b207-probe2	TGCACAGCTGCTCAGATG/3BioTEG
mPpp1r1b207-probe3	AGCCGAGGAAAGGAACAGTGT/3BioTEG
mNeat1 P1	TACCATCAGCCTTTAGATTT/3BioTEG
mNeat1 P2	CCCATTCAACAGTGAAGCAA/3BioTEG
mNeat1 P3	CTTCACTTCTTGGCAATCAG/3BioTEG

Table S4. Coordinates and lengths of Ppp1r1b-lncRNA deletion constructs used in RNA pulldown assays

Construct	Description	Coordinates (nt)	Length (nt)
WT	Full-length transcript	1–435	435
Del_1_100	Δ1–100 (nt)	101–435	335
Exon1_2	Contains Exons 1–2 only	1–220	220
Exon3_4	Contains Exons 3–4 only	221–435	215
Exon1_2_3	Contains Exons 1–3 only	1–343	343
Negative control antisense	Antisense of WT full-length transcript	1–435	435

Coordinates refer to the full-length sequence (Ensembl ENSMUST00000152525.2).

Supplemental Figure Legends

Fig. S1. Positive controls validating CHIRP, CHIP, RIP and RNA pulldown assays. (**A**) Validation of the CHIRP assay using Neat1 as a positive control. (**B**) Validation of CHIP assay using H3K27me3 enrichment at the Hoxa9 locus. (**C**) Validation of the EZH2 RIP assay in C2C12 cells. Enrichment of Neat1 RNA, a known EZH2-associated LncRNA, was measured by qPCR. (**D**) Validation of the RNA pull-down assay in hSMPC cells. (**E**) Biotinylated RNA pull-down was performed using AR RNA as a positive control and poly(A)25 RNA as a negative control. Pulled-down proteins were analyzed by immunoblotting for HuR, a known AR RNA-binding protein. GM: Growth media; DM: Differentiation Media. Data are presented as mean ± SEM from n = 3 independent biological replicates. Statistical significance was determined using an unpaired two-tailed Student's t-test. *$P < 0.05$; **$P < 0.01$.

Fig. S2. Validation of the hnRNPA1 knockdown by RNAi. (**A**) hnRNPA1 Western blot, replicate 1. (**B**) hnRNPA1 RNAi western blot, replicate 2 and 3. Representative blots from three independent experiments are shown. (**C**) Quantification of hnRNPA1 protein levels normalized to the corresponding loading control, β–actin or β-tubulin. Data represent mean ± SD from three independent experiments. Statistical significance was determined using an unpaired two-tailed Student's t-test. *P* value: **≤ 0.01; *≤ 0.05.

Fig. S3. hnRNPA1 detection in Cell lysate (input) and RNA pulldown complexes. (**A**) Western blot analysis of hnRNPA1 expression in undifferentiated (GM) and differentiated (DM) C2C12 cell lysates. β-Actin was used as a loading control. Right panel shows densitometric quantification of hnRNPA1 levels in cell lysate. (**B**) RNA pull-down assays were performed using biotin-labeled Ppp1r1b-lncRNA in GM and DM cells. A scrambled RNA probe (−) was used as a negative control. Western blot analysis shows enrichment of hnRNPA1 in Ppp1r1b-lncRNA pull-down samples compared with the negative control. Representative blots from three independent experiments are shown. Right panel shows densitometric quantification of hnRNPA1 levels in RNA pulldown samples. Quantification data are presented as mean ± SD from three independent experiments. Statistical significance was determined using an unpaired two-tailed Student's t-test. *P* value: **≤ 0.01; *≤ 0.05. Uncropped blots are shown.

Fig. S4. RNA pull-down analysis of proteins associated with *Ppp1r1b-lncRNA*. (**A**) RNA pull-down assays were performed using biotin-labeled Ppp1r1b-lncRNA in undifferentiated (GM) and differentiated (DM) C2C12 cells. Western blot analysis shows the association of hnRNPA3 with Ppp1r1b-lncRNA compared with the negative control RNA. Lower Right panel shows densitometric quantification of hnRNPA3 levels in input lysates and Pull-down samples. Quantification data are presented as mean ± SD from three independent experiments. Statistical significance was determined using an unpaired two-tailed Student's t-test. *P* value: **≤ 0.01; *≤ 0.05. (**B**) RNA pull-down assays demonstrating association of hnRNPH2 with Ppp1r1b-lncRNA under GM and DM conditions. (**C**) RNA pull-down assays demonstrating association of hnRNPU with Ppp1r1b-lncRNA. GM: Growth media; DM: Differentiation Media. Representative blots are shown. Due to limited replicates, data in (B) and (C) are presented as qualitative observations.

Fig. S5. Qualitative Domain mapping of *Ppp1r1b-lncRNA* regions associated with EZH2 and hnRNPA1. Full-length mouse *Ppp1r1b-lncRNA* gene was dissected and in vitro transcribed in different combination as bait for RNA pulldown assay. The precipitated proteins were fractionated by SDS-PAGE and subjected to western blotting for EZH2 and hnRNPA1. Representative uncropped blots are shown.

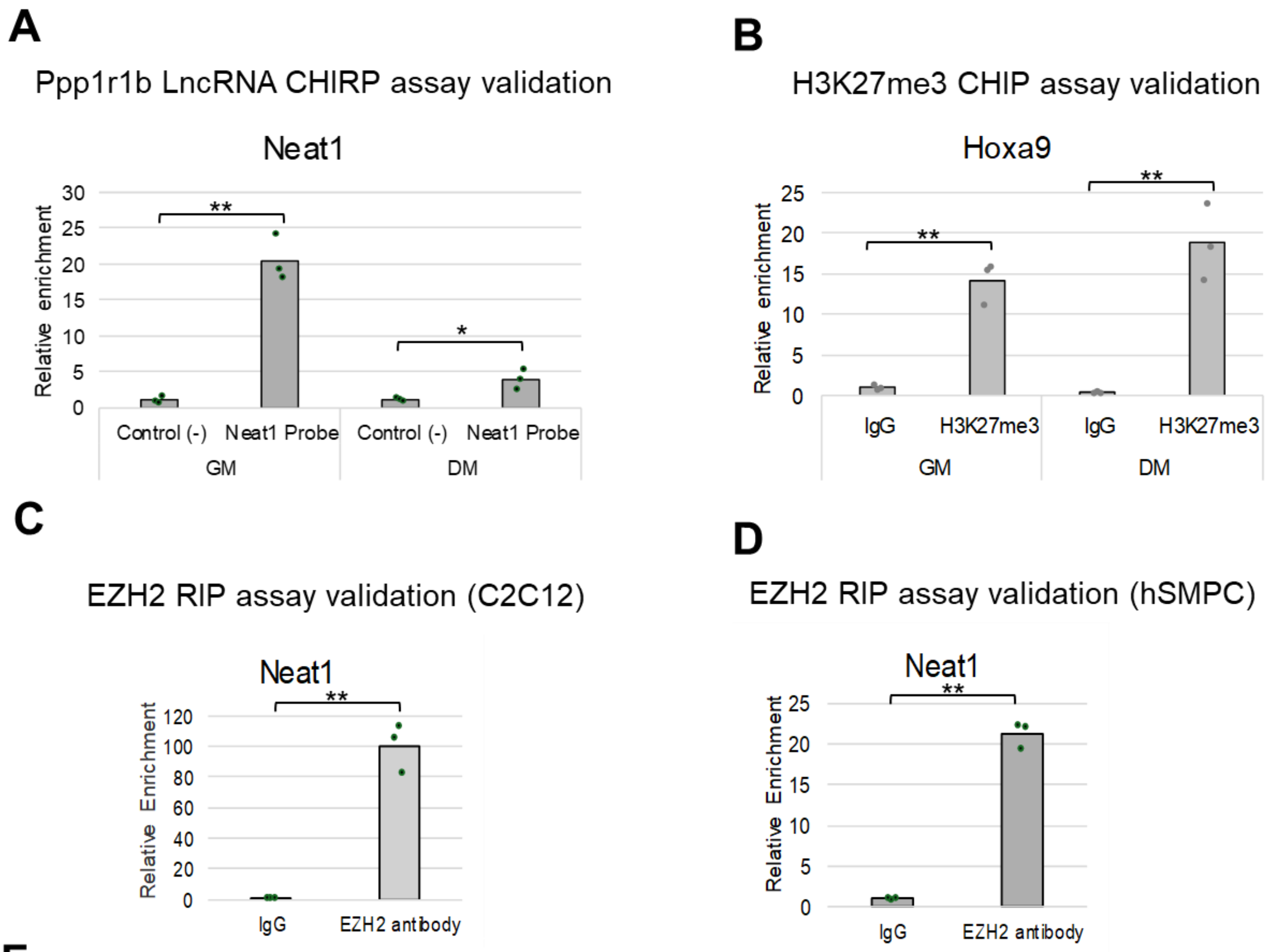

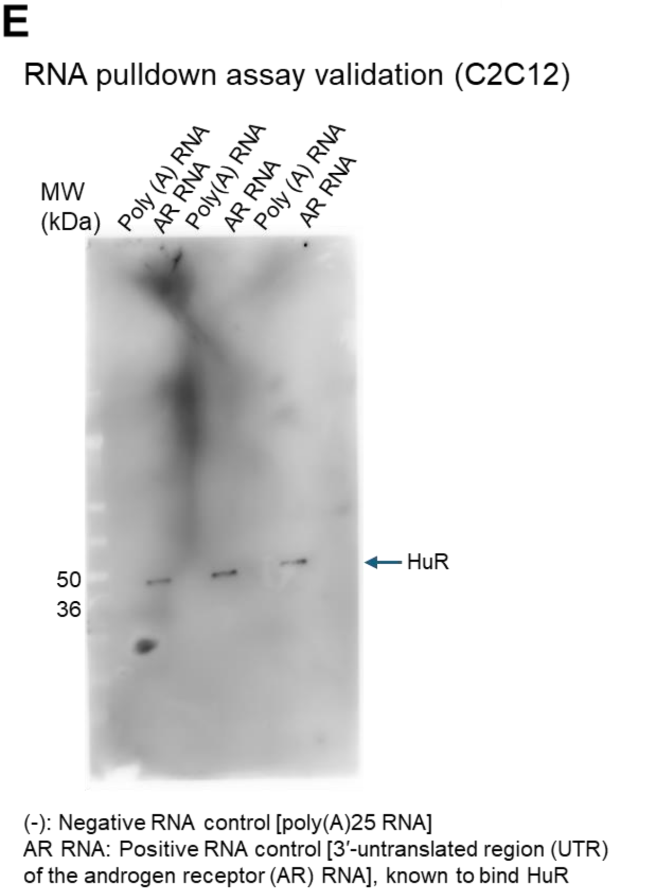

Fig. S1. Positive controls validating CHIRP, CHIP, RIP and RNA pulldown assays. (**A**) Validation of the CHIRP assay using Neat1 as a positive control. (**B**) Validation of CHIP assay using H3K27me3 enrichment at the Hoxa9 locus. (**C**) Validation of the EZH2 RIP assay in C2C12 cells. Enrichment of Neat1 RNA, a known EZH2-associated LncRNA, was measured by qPCR. (**D**) Validation of the RNA pull-down assay in hSMPC cells. (**E**) Biotinylated RNA pull-down was performed using AR RNA as a positive control and poly(A)25 RNA as a negative control. Pulled-down proteins were analyzed by immunoblotting for HuR, a known AR RNA-binding protein. GM: Growth media; DM: Differentiation Media. Data are presented as mean ± SEM from n = 3 independent biological replicates. Statistical significance was determined using an unpaired two-tailed Student’s t-test. *P < 0.05; **P < 0.01.

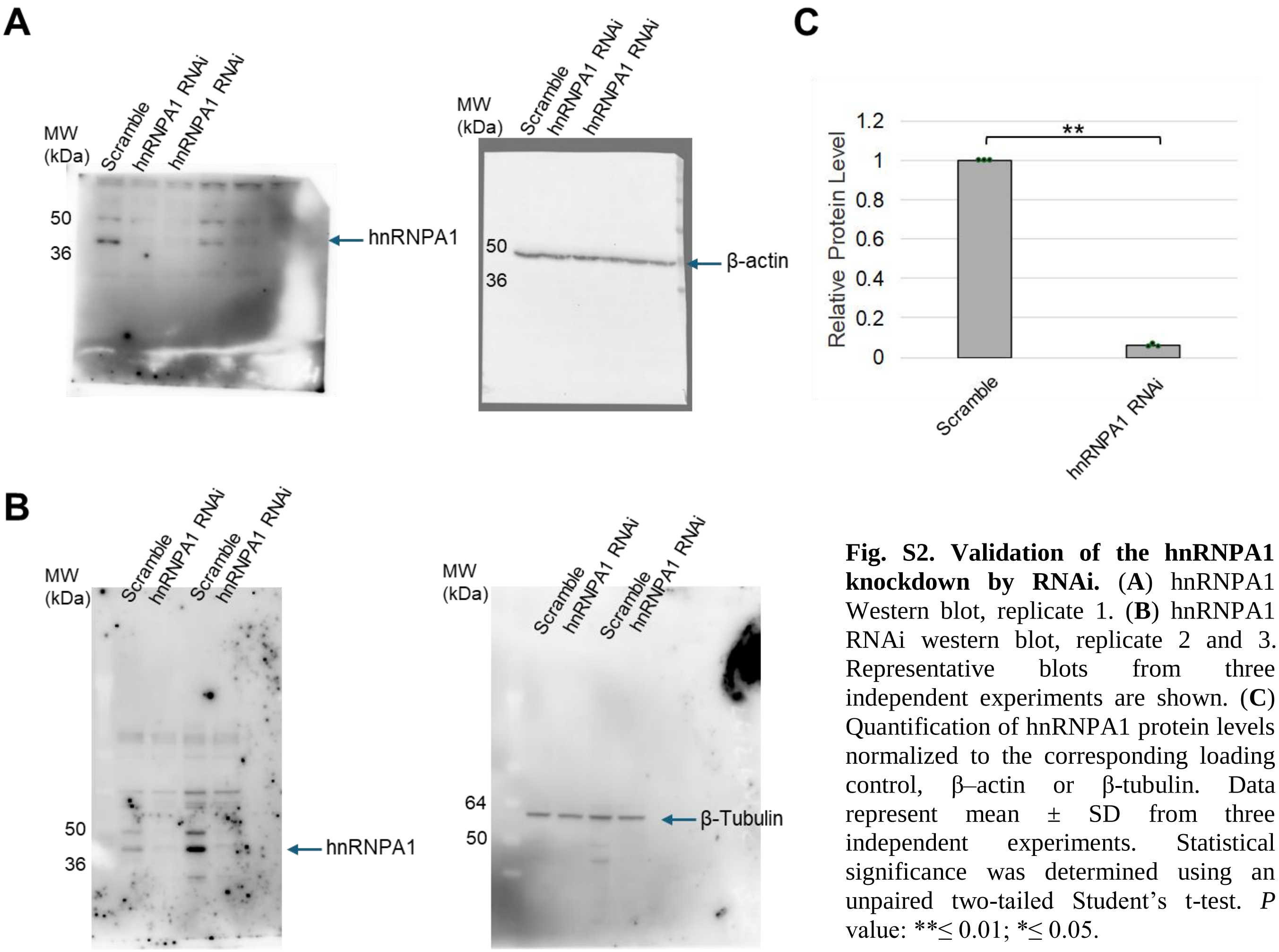

Fig. S2. Validation of the hnRNPA1 knockdown by RNAi. (**A**) hnRNPA1 Western blot, replicate 1. (**B**) hnRNPA1 RNAi western blot, replicate 2 and 3. Representative blots from three independent experiments are shown. (**C**) Quantification of hnRNPA1 protein levels normalized to the corresponding loading control, β–actin or β-tubulin. Data represent mean ± SD from three independent experiments. Statistical significance was determined using an unpaired two-tailed Student's t-test. *P* value: $** \leq 0.01$; $* \leq 0.05$.

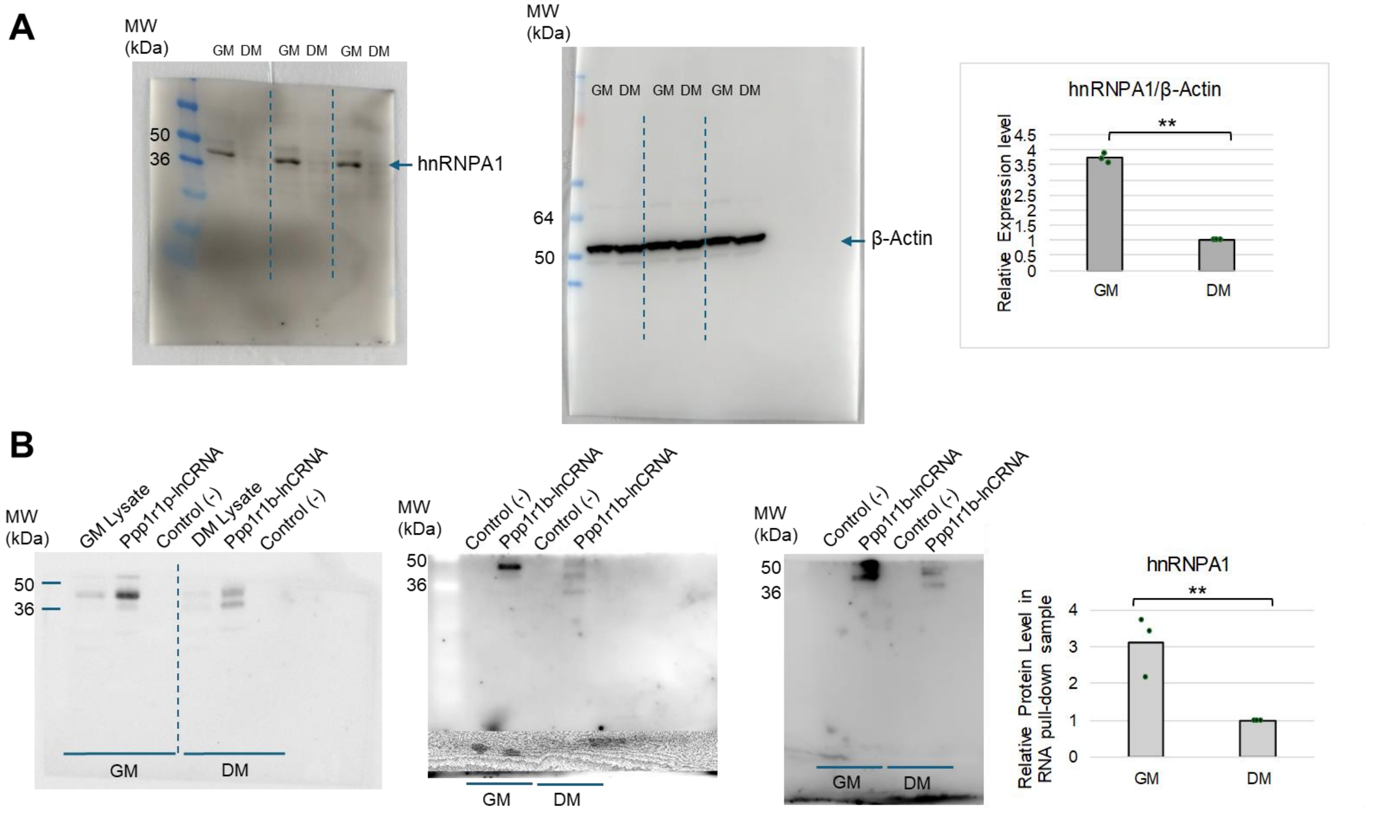

Fig. S3. hnRNPA1 detection in Cell lysate (input) and RNA pulldown complexes. (**A**) Western blot analysis of hnRNPA1 expression in undifferentiated (GM) and differentiated (DM) C2C12 cell lysates. β-Actin was used as a loading control. Right panel shows densitometric quantification of hnRNPA1 levels in cell lysate. (**B**) RNA pull-down assays were performed using biotin-labeled *Ppp1r1b-lncRNA* in GM and DM cells. A scrambled RNA probe (−) was used as a negative control. Western blot analysis shows enrichment of hnRNPA1 in *Ppp1r1b-lncRNA* pull-down samples compared with the negative control. Representative blots from three independent experiments are shown. Right panel shows densitometric quantification of hnRNPA1 levels in RNA pulldown samples. Quantification data are presented as mean ± SD from three independent experiments. Statistical significance was determined using an unpaired two-tailed Student's t-test. *P* value: **≤ 0.01; *≤ 0.05. Uncropped blots are shown.

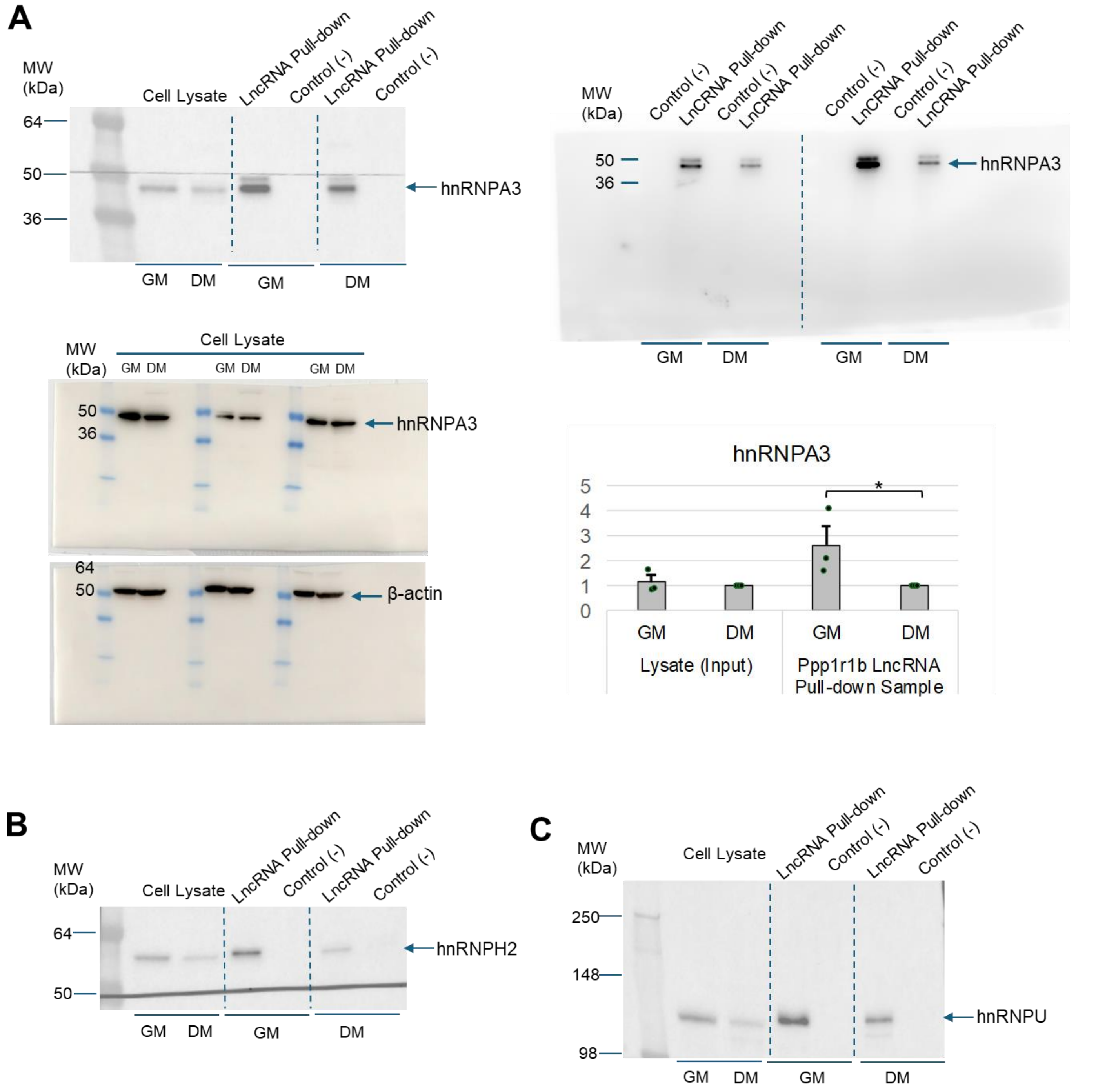

Fig. S4. RNA pull-down analysis of proteins associated with Ppp1r1b-lncRNA. (**A**) RNA pull-down assays were performed using biotin-labeled Ppp1r1b-lncRNA in undifferentiated (GM) and differentiated (DM) C2C12 cells. Western blot analysis shows the association of hnRNPA3 with Ppp1r1b-lncRNA compared with the negative control RNA. Lower Right panel shows densitometric quantification of hnRNPA3 levels in input lysates and Pull-down samples. Quantification data are presented as mean ± SD from three independent experiments. Statistical significance was determined using an unpaired two-tailed Student's t-test. *P* value: **≤ 0.01; *≤ 0.05. (**B**) RNA pull-down assays demonstrating association of hnRNPH2 with Ppp1r1b-lncRNA under GM and DM conditions. (**C**) RNA pull-down assays demonstrating association of hnRNPU with Ppp1r1b-lncRNA. GM: Growth media; DM: Differentiation Media. Representative blots are shown. Due to limited replicates, data in (B) and (C) are presented as qualitative observations.

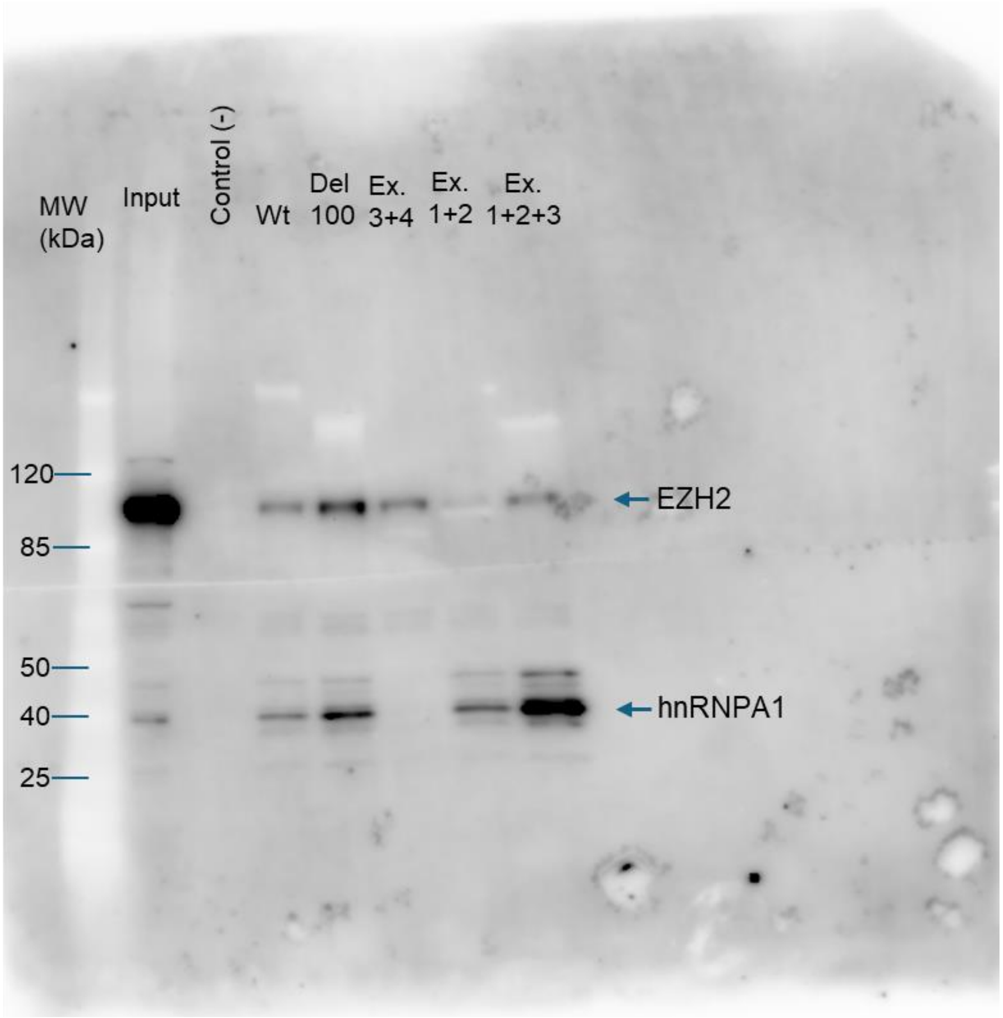

Fig. S5. Qualitative Domain mapping of *Ppp1r1b-lncRNA* regions associated with EZH2 and hnRNPA1. Full-length mouse *Ppp1r1b-lncRNA* gene was dissected and in vitro transcribed in different combination as bait for RNA pulldown assay. The precipitated proteins were fractionated by SDS-PAGE and subjected to western blotting for EZH2 and hnRNPA1. Representative uncropped blots are shown.
